# Redox Events As Modulators of Pathology and Therapy of Neuroinflammatory Diseases

**DOI:** 10.3389/fcell.2016.00063

**Published:** 2016-06-23

**Authors:** Klaudia Lepka, Carsten Berndt, Hans-Peter Hartung, Orhan Aktas

**Affiliations:** Department of Neurology, Medical Faculty, Heinrich-Heine University DüsseldorfDüsseldorf, Germany

**Keywords:** antioxidants, clinical trials, multiple sclerosis, redox signaling, oxidative stress

Neuroinflammation in the central nervous system (CNS) is characterized by increased production of chemokines and cytokines, altered integrity of the blood-brain-barrier, influx of leukocytes as well as the activation of microglia and astroglia. Although not all characteristics are present under the following conditions, stimuli eliciting a neuroinflammatory response can be toxins, infections, autoimmune reactions, traumatic injury, psychological stress, and epileptic seizures (Vezzani et al., 2011; Barnum et al., 2012; Xanthos and Sandkühler, 2014). Further, neuroinflammation has been linked to mechanisms of disease and clinical outcomes in neurodegenerative disorders like Alzheimer's and Parkinson's disease (Amor et al., 2010). In the following, we will mainly concentrate on Multiple Sclerosis (MS) as the prototype for an autoimmune inflammatory and degenerative disorder of the CNS. According to our current understanding, the immunopathogenesis of MS is as heterogeneous as its clinical manifestations and course and may be mediated by myelin-reactive T lymphocytes, leading to oligodendroglial cell death and demyelination, as well as to bystander axonal degeneration, neuronal loss and, finally, gliosis (Hartung et al., 2014). B cells may have a fundamental role in presenting antigens to T cells and as a consequence trigger an aberrant T cell response. Moreover, upon differentiation into plasmablasts and plasma cells that manufacture antibodies (Yuseff et al., 2013; Nutt et al., 2015), they may induce demyelination through antibody-mediated complement activation (Holers, 2014). Of note, while remyelination may occur in early stages of disease, regeneration is severely compromised as the disease progresses (Kremer et al., 2016). However, the etiology and cause for disease progression and failure of recovery remain largely elusive. Regarding possible factors, reactive oxygen (ROS), and nitrogen species (RNS) have attracted increasing interest in the last two decades. Focusing on MS we will discuss the role of ROS and RNS in disease onset and progression of this disabling disease and further emphasize the role of specific redox signaling modulating protein activity and its underestimated role in the development of new therapeutic agents.

## Oxidative and nitrosative stress in multiple sclerosis onset and progression

**Figure 1:**
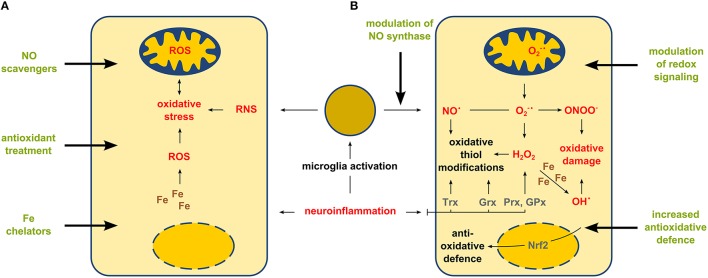
**Gross (unspecific) treatment of oxidative stress vs. targeted modulation of enzyme-based thiol redox modifications**. Neuroinflammation affects CNS cells by increasing the amounts of ROS and RNS. Panel **(A)** shows a simplified view on intracellular effects and therapeutic strategies aiming in the overall change of the amount of ROS and RNS. Panel **(B)** shows a more detailed explanation of cellular redox responses during neuroinflammation and potential therapeutic strategies aiming in the control of specific enzyme-based redox events mediated by nitric oxide synthase, transcription factors (such as Nrf2) or oxidoreductases thioredoxin (Trx), glutaredoxin (Grx) and peroxiredoxin (Prx) as well as other antioxidant enzymes, e.g., glutathione peroxidase (GPx).

The onset of MS is characterized by inflammation-mediated demyelination due to lymphocyte infiltration from the peripheral blood and microglial activation *in situ*. Subtle signs of neurodegeneration are identifiable from the beginning, characterized by axonal transection within white matter lesions (Trapp et al., 1998; Kuhlmann et al., 2002). This is of clinical importance particularly in chronic stages of disease, when extended cortical demyelination occurs which in aggregate represent the pathological substrate of permanent neurological disability (Zipp and Aktas, 2006). In both disease stages accumulation of ROS and RNS has been observed (Carvalho et al., 2014). In this context, one has to consider that the terms “ROS” and “RNS” summarize a variety of molecular species which substantially differ in chemical nature, cellular localization, and biological function (Figure 1). Unfortunately, these recent advances in our understanding of redox biology still go unrecognized by many researchers.

The majority of these molecular species are non-radicals: All oxygen radicals are ROS, but not all ROS are oxygen radicals. Moreover, depending on the origin, cellular functions of the respective oxygen radicals could be even oppositional (Prozorovski et al., 2015). Nevertheless, increased ROS levels are a prerequisite for and a consequence of oxidative stress. Per definition, oxidative stress is an imbalance between oxidants and antioxidants in favor of the oxidants, leading to a disruption of redox signaling and control and/or molecular damage (Sies and Jones, 2007). Clearly, such an imbalance does not imply a change in the overall cellular redox state. There is no general redox state of a given cell, although some researchers still use the Nernst equation and the glutathione: glutathione disulfide redox couple to determine a cellular redox state. However, this concept ignores all other redox couples, the compartmentalization of redox potentials, and the issue that glutathione requires enzymes to exert its biological functions (Flohé, 2013; Berndt et al., 2014). In the CNS, activated immune cells like microglia are a major source of reactive species. The neural parenchyma in the CNS is highly sensitive to oxidative damage, DNA double strand breaks, membrane disruption and protein degradation, due to its high cellular metabolic activity and enrichment in polyunsaturated fatty acids (Bazinet and Layé, 2014). Further, amounts of antioxidant molecules like α-tocopherol and antioxidant enzymes like superoxide dismutases (SOD), catalase, or glutathione peroxidases (GPx) are decreased in the brain compared to other tissues (Dringen, 2000; Chiurchiù et al., 2016). These CNS-specific characteristics might reinforce mitochondrial DNA damage based on pathological accumulation of reactive species which has been invoked as a possible reason for chronic neurodegeneration as well as for failure of remyelination (Li et al., 2005; Campbell et al., 2014; Witte et al., 2014). Extensively secreted nitric oxide (NO) reacts rapidly with ${\text{O}}_{2}^{-\cdot }$ forming ONOO^−^ and by this induces protein nitration in lesion areas. Nitrotyrosine is considered a hallmark of oxidative damage in neurodegenerative diseases (Pacher et al., 2007). Furthermore, iron accumulation in lesion areas promote oxidative damage of proteins, lipids, and nucleotides (Hametner et al., 2013). In summary, oxidative stress is considered as a major contributor to neuroinflammatory diseases including MS (Haider, 2015; Mahad et al., 2015). However, the direct and specific contribution of ROS and RNS to disease progression still remains elusive.

## Current therapeutics in multiple sclerosis

The onset of MS is most commonly characterized by a relapsing remitting disease form (RRMS) which later progresses into a secondary progressive form (SPMS). Disease-modifying treatments (DMTs) for RRMS are known to prevent or reduce the frequency of harmful immune responses targeted to CNS antigens and thereby slow or halt progression of disease pathology and accrual of neurologic disability. The implementation of easy-to-use magnetic resonance imaging (MRI)-guided proof of concept studies has paved the way for regulatory approval of 12 DMTs for RRMS including first-line medications like INFβ and glatiramer acetate, dimethyl fumarate (DMF) as well as teriflunomide and second-line options like natalizumab and alemtuzumab (humanized monoclonal antibodies), fingolimod and the immunosuppressant mitoxantrone (Fox, 2006; Ingwersen et al., 2016). Thereby, currently available therapeutics act mainly by modulating disease-relevant early immune activation steps, but fail to address repair of already damaged brain and spinal cord areas. Moreover, molecular mechanisms of the mode of action are still not entirely known for some of these drugs. However, more recently the mechanisms of function were investigated in greater detail underlining additional neurobiological effects of several DMTs, for instance fingolimod (Foster et al., 2007), as first-in-class spingosine-1-phosphate receptor modulator (Ingwersen et al., 2012) and DMF (Dubey et al., 2015). The molecular mechanism of DMF links this drug to prevention against oxidative stress (Albrecht et al., 2012).

The identification of oxidants and antioxidants involved in disease processes raised the hope that treatment with antioxidants could combat diseases connected to oxidative stress. In the last two decades a variety of clinical studies were initiated to test the impact of antioxidant donation itself and as adjunct medication in RRMS. Surprisingly, the majority of those studies failed (Gilgun-Sherki et al., 2004; Carvalho et al., 2016) although the respective compounds such as lipid peroxyl scavengers (Hall, 1992), low molecular weight antioxidants (Hansen et al., 1995), and others showed to some extent an influence on the progression of inflammation in cell culture or animal models (Chiurchiù et al., 2016). The failure of such clinical studies might be explained by the hitherto neglected roles of specific ROS, especially H_2_O_2_ and NO, as important second messengers in cellular signaling. Thus, excess of antioxidants does not just attenuate oxidative stress, but could also interfere with anti-inflammatory response (Ohl et al., 2016) and with physiological redox signaling and thus harmfully impact recovery processes.

## Redox signaling

During recent years, redox signaling, and redox regulation emerged as one of the major physiological control mechanisms in all yet investigated cell types. Redox signaling is even a regulator of other well-established and accepted signaling pathways, e.g., phosphorylation (Corcoran and Cotter, 2013), and can affect signaling by regulation of transcription factors or enzymatic activities via thiol modifications. Thiols can undergo several reversible oxidative posttranslational modifications, e.g., nitrosylation, glutathionylation, formation of disulfides, and sulfenic acid. Key enzymes in thiol redox regulation are oxidoreductases of the thioredoxin family, namely thioredoxins (Trx), glutaredoxins (Grx), and peroxiredoxins (Prx) (Hanschmann et al., 2013; Lillig and Berndt, 2013), which display cell type specific expression in the rat CNS (Aon-Bertolino et al., 2011) and catalyze the reduction and oxidation of specific cysteinyl residues and the intracellular level of the second messenger H_2_O_2_. Another protein regulating the amount of H_2_O_2_ is GPx (Deponte, 2013).

Redox regulation of transcription is well established (Brigelius-Flohé and Flohé, 2011). Very important in defense against oxidative damage is Nuclear Factor-E2-related factor 2 (Nrf2), a transcription factor controlling the transcription of several antioxidant enzymes. Activity of Nrf2 itself is regulated by the thiol redox state of Kelch-like ECH associated protein 1 (Keap1). In its reduced state, Keap1 promotes ubiquitination and subsequent degradation of Nrf2. Oxidized Keap1 allows the accumulation of Nrf2 in the nucleus and the expression of its target genes. Keeping this in mind, important redox events induced by the formation of reactive species during disease onset and progression might be simplified as oxidative or nitrosative stress. To date, it is not clarified whether redox changes may have different roles according to disease stage. Obviously, during CNS inflammatory attacks, invading lymphocytes, and activated macrophages/microglia initiate an acute and massive ROS/RNS challenge of the tissue characterized by damage of proteins, lipids, and nucleotides and thereby leading to immediate structural demise. In contrast, mild but persistent exposure to inflammation—as found in post-acute/chronic progressive stage—may result in alteration of specific redox regulation accompanied by targeted modification of redox-sensitive signaling pathways.

## Consequences for future therapies

Increased knowledge of enzyme-based redox events involved in disease onset and progression as well as potential redox-related modes of action of already existing drugs might pave the way for new therapeutic strategies, even approaches targeting regeneration in MS. For instance, preclinical studies revealed antioxidant properties of DMF acting via the translocation of Nrf2 into the nucleus and thereby promoting defense mechanisms against oxidative damage (Albrecht et al., 2012). Thereby, DMF treatment attenuates neuroinflammation and affects progression of MS and other neurodegenerative diseases (Johnson and Johnson, 2015; Buendia et al., 2016). It has been proposed that this mechanism is also the reason for the recently discovered neuroprotective and myelin-protective functions of DMF (Dubey et al., 2015). Of note, Nrf2 is upregulated in active MS lesions (Licht-Mayer et al., 2015). So far, the number of studies investigating the role of oxidoreductases or other antioxidant enzymes during MS is very limited, although these proteins are important during inflammatory processes, e.g., activation of macrophages (Salzano et al., 2014). Activity of GPx is dramatically decreased in cerebrospinal fluid and in serum of MS patients (Calabrese et al., 1994; Socha et al., 2014). In contrast, Prx5 as well as the mitochondrial oxidoreductases Trx2 and Prx3 are upregulated within MS lesions (Holley et al., 2007; Nijland et al., 2014) and Prx6 is increased in the spinal cord of mice that underwent experimental autoimmune encephalomyelitis, a common animal model of MS (Yun et al., 2015). Transgenic mice overexpressing Prx6 displayed attenuated blood-brain barrier leakage and neuroinflammation after induction of this model. Besides novel potent anti-inflammatory therapies, regeneration might be achieved by enzyme-based thiol redox modulation: Collapsin response mediator protein 2 (CRMP2) was proposed as a potential novel drug target for axonal regeneration after neuroinflammation (Petratos et al., 2010). Interestingly, CRMP2 mediated axonal outgrowth depends on redox regulation via Grx2 and Trx1 (Bräutigam et al., 2011; Morinaka et al., 2011).

Thus, instead of unspecific application of ROS scavengers or other broadly active antioxidants, therapies aiming at the specific modulation of enzyme-based redox regulation and signaling might be the promising future of what is called “redox medicine” (Figure 1) (Sies, 2015).

## Conclusion

In summary, recent insights have fundamentally changed our understanding of disease-related redox processes. Obviously, the unspecific use of the term “oxidative stress” has competed with latest insights indicating that subtle changes of the redox status of single molecules have a profound effect on both, endogenous signaling pathways relevant for inflammation as well as neuroregeneration. Basic as well as translational and clinical research in this area should consider these recent shifts in paradigms regarding oxidative stress, cellular redox potentials, ROS and RNS, and redox signaling.

## Author contributions

All authors listed, have made substantial, direct and intellectual contribution to the work, and approved it for publication.

### Conflict of interest statement

The authors declare that the research was conducted in the absence of any commercial or financial relationships that could be construed as a potential conflict of interest.
